# Pre- and Post-COVID-19 Antimicrobial Resistance Pattern of Pathogens in an Intensive Care Unit

**DOI:** 10.3390/ph17040407

**Published:** 2024-03-22

**Authors:** Andreea-Loredana Golli, Ovidiu Mircea Zlatian, Monica Laura Cara, Mădălina Olteanu

**Affiliations:** 1 Department of Public Health and Management, University of Medicine and Pharmacy of Craiova, 200349 Craiova, Romania; andreea_golli@yahoo.com; 2 Department of Microbiology, University of Medicine and Pharmacy of Craiova, 200349 Craiova, Romania; 3 Department of Orthodontics, University of Medicine and Pharmacy of Craiova, 200349 Craiova, Romania

**Keywords:** antimicrobial resistance, COVID-19, pandemic, pathogens

## Abstract

We aimed to determine the trend of the antimicrobial resistance pattern of pathogens isolated in samples collected from patients hospitalized in the intensive care unit (ICU) in selected periods before and after COVID-19. A retrospective study of bacterial pathogens was performed on 1267 patients. Positive bacterial culture data from 1695 samples from the pre-COVID-19 period and 1562 samples from the post-COVID-19 period were obtained. The most frequently isolated bacteria in both periods were *Staphylococcus aureus* and *Klebsiella* spp. The resistance rates of *Klebsiella* spp. Significantly increased against colistin (0.38% to 20.51%), gentamicin (44.62% to 64.85%), and aztreonam (56.35% to 3.60%). There was a significant increase in the resistance rate against colistin for *E. coli* strains (4.69% to 32.46%) and for Acinetobacter sp. strains (3.37% to 18.09%). More than 50% of the *Staphylococcus aureus* strains were MRSA, with statistically significant increases in the antimicrobial resistance rate against doxycycline (40.08% to 51.72%), linezolid (0.22% to 3.13%), rifampicin (53.16% to 64.93%), and teicoplanin (26.31% to 53.40%). The study revealed a significantly increasing trend in the antimicrobial resistance rate of Gram-negative pathogens against certain antibiotics, including those used only in cases where there are no other therapeutic options.

## 1. Introduction

Antimicrobial resistance (AMR) is one of the world’s most important public health issues, being one of the top 10 global public health threats facing humanity, according to WHO [1]. In 2019, it was estimated that 1.27 million deaths were attributable to bacterial AMR [2], with multidrug-resistant organisms being significantly associated with more economic burdens than susceptible organisms or those without infection or colonization [3].

The COVID-19 pandemic has been a major public health problem that has suddenly emerged with negative implications for all health systems worldwide. Due to the empirical prescription of antibiotics to many COVID-19 patients to prevent secondary bacterial infections, this is likely to lead to increase antimicrobial resistance. The emergence of bacterial pathogens that became tolerant to alcohol-based sanitizers is added to this [4,5].

According to ECDC, in the EU/EEA in 2020 alone, more than 800,000 cases of infections with antibiotic-resistant bacteria that are under European Antimicrobial Resistance Surveillance Network (EARS-Net) surveillance were diagnosed, and over 35,000 people died because of these infections. In 2022, the highest percentages and estimated incidence of bloodstream infections with resistant bacteria were reported by countries in the south and east of Europe [6].

In 2020 and 2021, within the COVID-19 epidemic that evolved in Romania as part of the pandemic, 1,813,823 cases and 58,971 deaths were registered (general fatality rate = 3.3%) [7]. In 2022, 1,502,755 cases were reported (incidence rate = 7857/100.000) and 8653 deaths (mortality rate = 45/100.000; fatality rate = 0.58%). Compared to 2021, the number of cases decreased by 5% and the number of deaths by 80% [7].

Today, COVID-19 is no longer a global emergency, but it is currently estimated that approximately 700,000 people die because of AMR each year, and, by 2050, the number of deaths is expected to rise to 10 million deaths every year [8].

One of the strategic objectives of the Global Action Plan on Antimicrobial Resistance is to know the incidence, prevalence, and pattern of AMR by pathogen categories, understand the mechanisms of development and spread of resistance, and have the ability to rapidly characterize new emerging types of resistance [9].

Consequently, studies assessing the impact of the COVID-19 pandemic on AMR are needed to improve healthcare, especially for critically ill patients hospitalized in intensive care units (ICUs) who require intensive antibiotic treatment and invasive procedures.

Our research aims to determine the trend of antimicrobial resistance pattern of pathogens isolated in samples collected from patients hospitalized in the ICU, in selected periods before and after COVID-19.

## 2. Results

**Figure 1:**
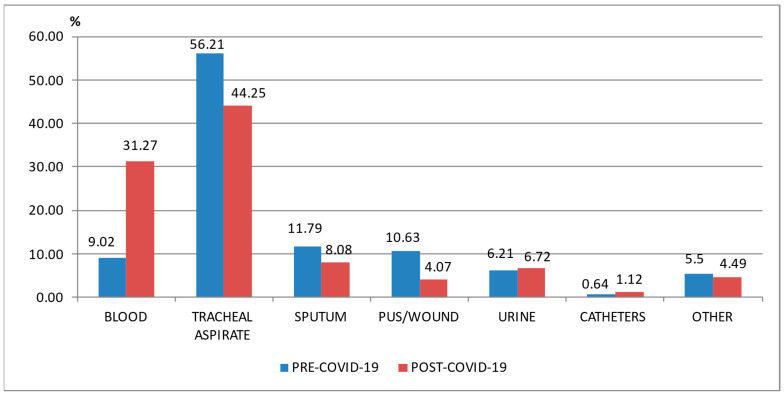
Distribution of samples from patients hospitalized in the ICU, County Emergency Clinical Hospital Craiova, Romania, pre- and post-COVID-19 era.

In this research, 3257 samples were collected. A total of 1562 samples and 1940 bacterial isolates (Figure 1) were obtained in the pre-COVID-19 period from 1267 patients admitted to the ICU (797 males—62.90%; 470 females—37.10%; average age: 64 ± 18.60) (Table 1). A total of 1695 samples and 2281 bacterial isolates were obtained from 1354 patients in the post-COVID-19 period (841 males—62.11%; 513 females—37.89%; average age: 64 ± 17.51).

In the pre-COVID-19 period, most samples came from the respiratory tract (1062—68%), pus/wound swabs (166—10.63%), and blood (141—9.02%). In the post-COVID-19 period, almost half of the samples originated from the respiratory tract (887—52.33%), while the number of blood samples was more than three times higher compared with the pre-COVID-19 period (530—31.27%). The number of pus/wound swabs from which pathogens were isolated was less than half in the post-COVID-19 period (69—4.07%) compared to the pre-COVID-19 period (166—10.63%) (Table 2).

**Figure 2:**
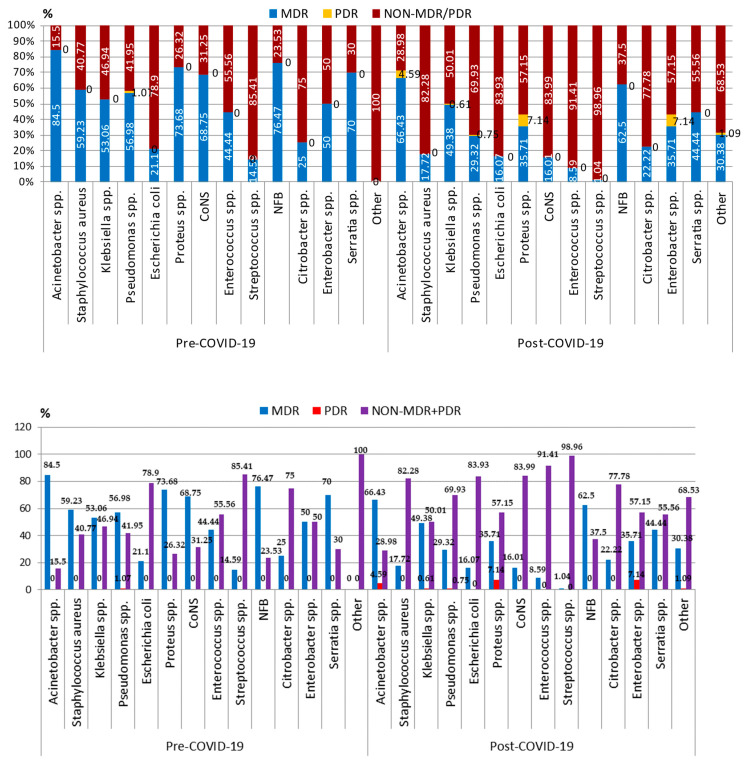
Distribution of the micro-organisms isolated from samples from patients hospitalized in the ICU, County Emergency Clinical Hospital Craiova, Romania, pre- and post-COVID-19 periods. NFB—Nonfermenting Gram-negative bacilli; CoNS—Coagulase-negative staphylococci; MDR—Multidrug-Resistant; PDR—Pan-Drug-Resistant.

The most frequently isolated of all micro-organisms identified in the harvested samples in both the pre-COVID-19 and post-COVID-19 period were *Staphylococcus aureus* and *Klebsiella* spp. (Figure 2).

In the pre-COVID-19 period, the most commonly reported Gram-negative bacterial pathogen registered was *Klebsiella* spp. (343—17.68%), followed by *Acinetobacter* spp. (213—10.98%), Proteus (133—6.86%), *E. coli* (109—5.62%), and *Pseudomonas* spp. (93—4.80%). More than half of the strains (1041—53.66%) were multidrug-resistant (MDR). (Figure 2). The most prevalent Gram-positive bacterial pathogen was *Staphylococcus aureus* (*S. aureus*) (493—25.41%), followed by *Streptococcus* spp. (281—14.48%). A total of 315 strains of *S. aureus* were methicillin-resistant (MRSA—methicillin-resistant *Staphylococcus aureus*).

In the post-COVID-19 period, the hierarchy was preserved in the case of Gram-negative bacterial pathogens, but the percentage of strains of *Acinetobacter* spp. (12.41%—283), *E. coli* (168—7.36%), and *Pseudomonas* spp. (133—5.83%) increased, while that of *Klebsiella* spp. decreased (14.29%—326). In the case of Gram-positive pathogens, there was an important increase in the number and percentage of the strains of Coagulase-negative staphylococci (CoNS) (256—11.22%) and *Enterococcus* spp. (128—5.61%), while those of *Staphylococcus aureus* and *Streptococcus* spp. were lower.

Almost 70% of all the pathogens identified in the samples harvested in the pre-COVID-19 period and almost 60% in the post-COVID-19 period were found in tracheal aspirate/sputum (Figure 1), and *Staphylococcus aureus* and *Klebsiella* spp. were the most common isolated pathogens from these samples (Figure 1).

More than half of the isolated pathogens in blood samples were CoNS (55.19%) in the pre-COVID-19 period, followed by *Klebsiella* spp. (11.03%). In the post-COVID-19 period, CoNS were the most prevalent (40.13%), followed by *Acinetobacter* spp. (8.15%) and *Klebsiella* spp. (5.7%). The most prevalent pathogen in tracheal aspirate/sputum was *S. aureus*, both in the pre-COVID-19 (30.03%) and in the post-COVID-19 period (28.13%). In urine specimens, *Klebsiella* spp. (30.55%) was the most predominant pathogen in the pre-COVID-19 period, while E coli occupied the first place in the post-COVID-19 period (33.04%).

*S. aureus* held, in the pre-COVID-19 period, the highest percentage from pus/wound swabs among isolated pathogens (22.70%), while *Klebsiella* spp. was the most prevalent in the post-COVID-19 period, followed by *Acinetobacter* spp., *Proteus* spp., and *S. aureus* (12.90%). A small number of pathogens was isolated from intravascular catheters, *S. aureus* being the most prevalent in both periods (Table 2).

More than half of all the pathogens were MDR in the pre-COVID-19 period and almost a third in post-COVID-19 period. Among the MDR Gram-positive pathogens, almost 60% of the *S. aureus* strains (292/493), 70% of the CoNS strains (88/128), and 45% (20/45) of the *Enterococcus* spp. strains were MDR in the pre-COVID-19 period. In the post COVID-19 period, only 17.72% of the *S. aureus* strains (78/440) were MDR. Although the number of CoNS strains was almost double (256) in the same period, only 16% were MDR. The same thing was found in the case of strains of *Enterococcus* spp. (128 strains, 11 MDR) (Figure 2).

In the case of the most frequently isolated Gram-negative bacteria, a very high percentage of MDR isolates was found in the pre-COVID-19 period in the cases of *Acinetobacter* spp. (84.5%), Nonfermenting Gram-negative bacilli (NFB) (76.47%), *Klebsiella* spp. (53.06%), *Proteus* spp. (73.68%), and *Pseudomonas* spp. (56.98%) (Figure 2). Also, a pan-drug-resistant (PDR) Pseudomonas strain was isolated. One fifth of the *E. coli* strains (23/109) were MDR. Although in the post-COVID-19 period, there was a decrease in the number and percentage of Gram-negative strains, 16 strains were PDR, of which 13 were *Acinetobacter* spp. (Figure 2).

The antibiotic resistance rates of the Gram-positive and Gram-negative isolates are summarized in Table 3 and Table 4.

In the case of antimicrobial resistance rates of the Gram-negative bacteria, almost 80% from the *Klebsiella* spp. strains isolated in both periods were resistant to first-generation cephalosporins, around 65% to third-generation cephalosporins, and 55–60% to fourth-generation cephalosporins (Table 3). Overall, the number of isolated *Klebsiella* spp. strains decreased in the post-COVID-19 period, but the number of resistant strains was higher, with a statistically significant difference (*p* < 0.001) for Cefotaxime. Around 45% of the strains were resistant to carbapenems (imipenem and meropenem) and around 55% to fluoroquinolones, with no statistically significant difference between the two periods. For most of the tested antibiotics, there were no significant differences between the two periods. *Klebsiella* spp. had a statistically significant increase in resistance rates (*p* < 0.001) in the post-COVID-19 period, compared to the pre-COVID-19 period, against colistin (0.38% to 20.51%), gentamicin (44.62% to 64.85%), and aztreonam (56.35% to 3.60%). For piperacillin-tazobactam, a decrease was registered in the resistance rates, but without statistical significance. Two PDR Klebsiella strains were found (Figure 2).

In the case of *E. coli* strains, a significant increase was registered in the resistance rate against colistin (4.69% to 32.46%, *p* < 0.001) and a significant decrease against levofloxacin (52% to 28.76%, *p* = 0.03) and imipenem (16.49% to 6.14%, *p* = 0.01). In the case of all other tested antibiotics, no changes in resistance patterns pre- vs. post-COVID-19 were observed. The number of the isolated strains was higher in the post-COVID-19 period, with an increased resistance rate against amoxicillin/clavulanic acid, ceftazidime, cefotaxime, and meropenem, but without statistical significance (Table 3). The highest resistance rate was against cefazolin, over 50%. The percentage of MDR strains decreased in the post-COVID-19 period (16.07%).

Antimicrobial resistance in *Acinetobacter* spp. isolates revealed a very high level of resistance against third-generation cephalosporins (over 95%). High resistance was also found to carbapenems (around 90%), piperacillin-tazobactam (from 88% to 91%), fluoroquinolones (from 88% to 95%), and aminoglycosides (around 85%) (Table 3). In the post-COVID-19 period, the number of Acinetobacter sp. strains was higher, with a significant increase in the antimicrobial resistance rate against colistin (3.37% to 18.09%, *p* < 0.001) and a significant decrease against cefepime (95.65% to 84.43%, *p* < 0.001) and aztreonam (96.25% to 81.03%, *p* = 0.003). For all the other tested antibiotics, no significant differences were found between the two studied periods. All the isolated *Acinetobacter* spp. strains were found to be resistant to amoxicillin/clavulanic acid and cefazolin. Also, almost 5% of the *Acinetobacter* spp. strains were PDR.

In the case of *Pseudomonas* spp. strains, the number of the isolated strains was higher in the post-COVID-19 period, and the highest resistance rate in both periods was against cefazolin and amoxicillin/clavulanic acid (100%). An increase in the antimicrobial resistance rate against third-generation cephalosporins was found for ceftazidime (from 60.92% to 67.72%) and cefotaxime (from 75% to 81.82%), and a decrease for ceftriaxone (from 82.35% to 70%), without statistical significance. The antibiotic resistance rate against fourth-generation cephalosporins (cefepime) was significantly lower in the post-COVID-19 period (from 76.47% to 58.51%, *p* = 0.01). The carbapenem resistance rate also decreased, but without statistical significance. The lower resistance rate was against colistin, with a significant increase (*p* = 0.03) in the post-COVID-19 period (4.88%), compared to the pre-COVID19 period (0%) (Table 3). With the mentioned exceptions, no significant variation in antibiotic resistance to the tested antibiotics was recorded between the two studied periods. Although the percentage of the MDR *Pseudomonas* spp. strains was almost halved in the post-COVID-19 period, a PDR strain was isolated in both periods (Figure 2).

The research revealed, in the case of *Proteus* spp. strains, a high resistance rate against amoxicillin/clavulanic acid, first- and third-generation cephalosporins, fluoroquinolones, and gentamicin, but the rate was lower in the post-COVID-19 period, with the difference being statistically significant for cefazolin (from 94.12% to 84.43%, *p* = 0.03), cefepime (from 54.80% to 21.37%, *p* < 0.001), and gentamicin (from 76.59% to 55.97%, *p* < 0.001). This correlates with the decrease in the percentage of MDR strains, from 73.68% to 18.04%. The resistance rate against piperacillin/tazobactam was higher, but without statistical significance. All the tested strains were resistant to colistin.

Among the MDR Gram-positive pathogens, more than 50% of the *Staphylococcus aureus* strains were MRSA (315—63.89% in the pre-COVID-19 period; and 223—50.68% in the post-COVID-19 period). Almost 60% were MDR in the pre-COVID-19 period, but around 20% in the post-COVID-19 period. There were statistically significant increases in the antimicrobial resistance rate in the post-COVID-19 period against doxycycline (40.08% to 51.72%, *p* = 0.002), linezolid (0.22% to 3.13%, *p* < 0.001), rifampicin (53.16% to 64.93%, *p* < 0.001), and teicoplanin (26.31% to 53.40%, *p* = 0.03%). Significant decreases were found against erythromycin (74.17% to 55.58%, *p* < 0.001) and penicillin (98.34% to 87.01%, *p* < 0.001) (Table 4). No significant changes were found for the other tested antibiotics.

The number of isolated strains of CoNS was almost double in the post-COVID-19 period, but only 15% were MDR, compared to almost 70% in the pre-COVID-19 period (Figure 2). Over 90% of the strains were resistant to penicillin and 70% to tetracycline. High and increasing resistance rates were registered against clindamycin and clarithromycin, with no statistical significance. Significant increases were recorded in the post-COVID-19 period against rifampicin (45.24% to 62.55%, *p* = 0.001) and teicoplanin (26.67% to 63.57%, *p* < 0.001). In the same period, there was a significant decrease in the antibiotic resistance rate against erythromycin (from 80.65% to 68.27%, *p* = 0.02), vancomycin (from 15.79% to 0.91%, *p* < 0.001), and rifampicin (from 45.24% to 62.55%, *p* = 0.001). For all the other tested antibiotics, no changes in resistance patterns pre- vs. post-COVID-19 periods were found.

All *Streptococcus* spp. strains were resistant to ciprofloxacin in both periods. Half of the strains were MDR in the pre-COVID-19 period, reaching 60% in the post-COVID-19 period. A high resistance was observed against penicillin (85.11%), but it significantly decreased in the post-COVID-19 period (62.18%, *p* < 0.001). A significant decrease was also registered against oxacillin (from 91.89% to 34.15%, *p* < 0.001), erythromycin (from 52.55% to 29.11%, *p* < 0.001), and clarithromycin (from 49.12% to 27.03%, *p* < 0.001). The resistance rate of the *Streptococcus* spp. strains against doxycycline increased significantly, from 10.57% to 19.75%, *p* < 0.001. No other significant changes were found.

*Enterococcus* spp. resistance rate against penicillin was significantly higher in the post-COVID-19 period (61.22% compared to 34.15%, *p* = 0.01) and lower against doxycycline (80.65% to 33.33%, *p* < 0.001) and vancomycin (29.17% to 5.33%, *p* < 0.001) (Table 4). For all the other tested antibiotics, no significant changes were observed. The percentage of MDR strains was five times higher in the pre-COVID-19 period (44.44%), compared to the post-COVID-19 period (8.6%) (Figure 2).

## 3. Discussion

According to the European Antimicrobial Resistance Surveillance Network (EARS-Net) Report for 2022, AMR remains a concern in the EU/EEA, especially regarding the continuous increase in carbapenem-resistant *K. pneumoniae* and vancomycin-resistant E. faecium [6].

The COVID-19 pandemic has challenged health systems in all countries, with the reorganization of hospitals and limited access for patients with other conditions than COVID-19. Due to the lack of specific treatment, antibiotics have been empirically administered mainly to patients with severe forms of disease, and those hospitalized in the ICU. The inappropriate use of antibiotics in viral illnesses and in the absence of bacterial co-infection may be an enabling factor for the selection of multidrug-resistant strains. The overuse of antibiotics, especially those most used to treat secondary bacterial infections in COVID-19 patients (amoxicillin, azithromycin, and cephalosporins), has contributed to the development of new, antibiotic-resistant bacterial strains against them [10,11,12,13,14].

Romania has the second highest value in the EU/EEA of the indicator of antibiotic use with a major risk of selecting MDR/XDR bacteria: 55.1% compared to a European average of 38.6% [15].

Our research aims to determine whether the COVID-19 pandemic significantly changed the resistance of pathogens involved in cases of infection in hospitalized patients in the ICU—Emergency Clinical County Hospital of Craiova, in selected periods before and after COVID-19. To our knowledge, no similar studies have been published examining the possible changes in antimicrobial resistance in Romania, in the pre- and post-COVID-19 periods. Only one study investigated possible changes in uropathogen resistance in female patients before and during the COVID-19 pandemic [16,17]. The study found the greatest increase in resistance in *Klebsiella* spp. and Pseudomonas against quinolones, consistent with other research [18], and a significant increase in resistance to carbapenems. A decrease in resistance to penicillin was observed in *Enterococcus* spp.

There are several other studies that have found a relationship between COVID-19 and AMR, suggesting that some conditions, often including increased antibiotic usage, may be contributing to the rise of AMR, but most of them included for comparison the period before the pandemic and what happened during the pandemic.

A study conducted in India revealed an overall increase in carbapenem resistance rates between the pre-COVID-19 period and the COVID-19 period for *E. coli*, *Klebsiella pneumoniae*, *Acinetobacter baumanii*, and *Pseudomonas aeruginosa* [19].

Another study carried out in a University Hospital in Egypt showed a significant increase in XDR species during the COVID-19 era and, in the case of Gram-negative pathogens, a statistically significant increase in the resistance for ampicillin, ciprofloxacin, aztreonam, and cefazolin during the COVID-19 period compared with before the pandemic. The susceptibility pattern was not different from Gram-positive pathogens [20].

A significant decrease in resistance for carbapenem-resistant *Klebsiella pneumoniae*, *Pseudomonas*, and *Acinetobacter baumanii* during the pandemic compared with the pre-COVID-19 period, and a significant increase in the prevalence of vancomycin-resistant E. faecium were found in a research study conducted in Columbia [21].

According to a study conducted in Pakistan, *S. aureus* showed a decrease against oxacillin and erythromycin after the COVID-19 pandemic, with an increasing pattern of resistance in the case of *Enterococcus* spp. for ampicillin, gentamicin, and ciprofloxacin [22].

In our research, the most frequently isolated pathogens were *S. aureus* and *Klebsiella* spp. in both studied periods. CoNS were the most common pathogens involved in blood stream infections during both studied periods, while *S. aureus* was most commonly isolated from tracheal aspirate/sputum and intravascular catheters. *S. aureus* was most frequently identified also from pus/wound swabs in the pre-COVID-19 period, while in the post-COVID-19 period, Gram-negative pathogens prevailed. An increasing percentage of isolated Gram-negative strains was observed in the post-COVID-19 period (except for *Klebsiella* spp.), and of Gram-positive CoNS and *Enterococcus* spp. One third of urinary tract infections were caused by *Klebsiella* spp. in the pre-COVID-19 period and by *E. coli* in the post-COVID-19 period. These findings are consistent with those from other studies [23,24]. Both *Enterococcus* spp. and other negative pathogens are frequently implicated in the etiology of healthcare-associated infections, in European acute care hospitals [6].

A very high percentage of MDR isolates in the pre-COVID-19 period was found in the case of *Acinetobacter* spp., NFB, *Klebsiella* spp., *Proteus* spp., and *Pseudomonas* spp., with an important decrease in the post-COVID-19 period, both for Gram-negative and Gram –positive pathogens, but also with a significant increase in AMR to certain antibiotics.

AMR percentages for *Klebsiella* spp. had a statistically significant increase (*p* < 0.001) in the post-COVID-19 period compared with to the pre-COVID-19 period, against cefotaxime, colistin, gentamicin, and aztreonam. Around 50% of the strains were resistant to carbapenems (imipenem and meropenem) and to fluoroquinolones. These findings were consistent with the data registered at the national level, with carbapenem-resistant *K. pneumoniae* accounting for 47.5%, occupying second place among EARS Net states, and exceeding 4.75 times the European average [15]. The increasing resistance to backup antibiotics, such as colistin, is an issue of the greatest concern, which may be due to the use of these antibiotics in situations where they are not indicated, contributing to the selection of resistant pathogens. One of the few published studies evaluating changes in antimicrobial resistance [23] revealed a statistically significant decrease in the resistance of *Klebsiella* spp. against ceftazidime, colistin, and doxycycline, in the post-COVID-19 period compared to the pre-COVID-19 period. Another study, conducted in a COVID-19 referral hospital in Iran, showed a significant overall resistance increase among Gram-negative bacteria, particularly *P. aeruginosa* and *K. pneumoniae* [25]. A review conducted by Khaznadar et al. also revealed that Gram-negative pathogens were those with levels of antimicrobial resistance most affected by the overuse of antibiotics during the pandemic [26], while a retrospective study conducted in Greece showed an increasing trend in the incidence of resistant Gram-negative bacteria, particularly in ICUs, compared to the pre-pandemic period [27].

Antimicrobial resistance in *Acinetobacter* spp. isolates revealed a very high level of resistance against third-generation cephalosporins (over 95%), carbapenems (around 90%), piperacillin-tazobactam (around 90%), fluoroquinolones (around 90%), and aminoglycosides (around 85%). These findings are consistent with those from other studies [28,29,30,31] and with data on antibiotic consumption and antibiotic resistance in Romania, showing an increasing extended resistance (for *A. baumannii*, carbapenem resistance was 93.6% and multi-resistance was 89.4%, occupying third place for both indicators among EARS Net states) [15]. A significantly increasing trend was also observed in the antimicrobial resistance rate against colistin (*p* < 0.001), which is an issue of great concern because colistin remains one of the very few antibiotics which are consistently active against these bacterial species. A significant decrease was found against fourth-generation cephalosporins (*p* < 0.001) and monobactams (*p* = 0.003). In another research study [23], no statistically significant increase or decrease in resistance rates was observed between study periods for *Acinetobacter* spp.

In the case of *Pseudomonas* spp. strains, AMR decreased significantly in the post-COVID-19 period against fourth-generation cephalosporins (cefepime, *p* = 0.01) and increased against colistin (*p* = 0.03). An increasing trend was revealed against third-generation cephalosporins (ceftazidime and cefotaxime). The carbapenem resistance rate decreased, but without statistical significance. These results were different from another study [23], which revealed a significant decrease in the resistance against cefotaxime and meropenem. *P. aeruginosa* remains one of the major causes of healthcare-associated infection in Europe [6], and the increasing trend of resistance to certain antibiotics, including colistin, draws attention to the difficulty of treating infected patients, which is also proven by the identification of PDR *Pseudomonas* spp. strains during both studied periods. The rapid emergence of diverse resistant Gram-negative bacteria, including PDR, XDR (extensively drug-resistant), MDR, and carbapenem-resistant Gram-negative bacteria, due to the inappropriate consumption and prescription of antibiotics, has also been highlighted by other researchers [32].

A significantly decreasing trend was observed in the case of *Proteus* spp. strains resistant against first- (cefazolin, *p* = 0.03) and fourth-generation cephalosporins (cefepime (*p* < 0.001), and aminoglycosides (gentamicin *p* < 0.001), consistent with other findings [23]. An increase, but without statistical significance was found against piperacillin/tazobactam. The resistance against colistin was 100%, with Proteus being inherently resistant to this antibiotic.

In the case of *S. aureus* isolates, statistically significant increases in the antimicrobial resistance rate were found in the post-COVID-19 period against doxycycline (*p* = 0.002), linezolid (*p* < 0.001), rifampicin (*p* < 0.001), and teicoplanin (*p* = 0.03%), while significant decreases were found against erythromycin (*p* < 0.001) and penicillin (*p* < 0.001), a different trend compared to other researchers [23].

The number of the isolated strains of CoNS was almost double in the post-COVID-19 period. Significant increases were recorded in the post-COVID-19 period against rifampicin (*p* = 0.001) and teicoplanin (*p* < 0.001). In the same period, the antibiotic resistance rate against erythromycin (*p* = 0.02), vancomycin (*p* < 0.001), and rifampicin (*p* = 0.001) was significantly decreased. In another study [23], in the post-COVID-19 period, a significant decrease was found only against vancomycin.

The number of positive blood samples was more than three times higher In the post-COVID-19 period. Over 60% of *S. aureus* strains identified in blood samples were MRSA (19/34), less than from the pre-COVID-19 period (almost 80%, but higher than the percentage of invasive isolates resistant to methicillin reported in Romania (25–50%)) [6]. The same declining trend was seen in the EU/EEA population-weighted mean percentage of MRSA [6]. Instead, a study conducted in a University Hospital in Italy did not reveal a significant variation in the incidence density of bloodstream infections, except for an increase in those caused by carbapenem-susceptible Pseudomonas aeruginosa and in the trend of MRSA [33].

Almost 90% of *Klebsiella* spp. strains were carbapenem-resistant in the pre-COVID-19 period, compared to approximately 65% in the post-COVID-19 period. Although decreasing, the percentage was higher than that reported across the country (between 25% and 50%) [6].

Almost 25% of strains of *E. coli* responsible for bloodstream infections in the post-COVID-19 period were resistant to third-generation cephalosporins, compared to 50% of those isolated from blood samples in the pre-COVID-19 period. Although the study did not reveal significant changes in antimicrobial resistance for most tested antibiotics during the two periods, the percentage of MDR strains remains very high. The significant increase in Gram-negative pathogens resistance to the antibiotics used in cases without other therapeutic resources, like colistin, as well as the increase in the number of PDR strains, are worrying elements for the future evolution of AMR [34]. These findings are consistent with other studies, reflecting the negative impact of COVID-19 on patients with MDR-Gram negative bloodstream infections and on AMR inside ICU settings [25,35].

One of the possible causes of the selection of MDR pathogens is the unjustified prescription of antibiotics, including reserve antibiotics. During the COVID-19 pandemic, SARS-CoV-2 patients hospitalized in the ICU received, prophylactically or for secondary co-infections, third-generation cephalosporins and quinolones, which may contribute to the increased circulation of MDR pathogens. Another important issue is related to the status of patients admitted to ICUs, namely, critically ill patients, which often require connection to invasive devices. In order to stop the spread of multidrug resistance, doctors’ awareness of the need for a judicious prescription of antibiotics is essential, and the microbiological test should be obtained before the use of any antibiotics. Also, providing the necessary spaces and stringent isolation of patients infected with MDR micro-organisms, along with the adoption of infection prevention and control measures, such as hand hygiene and surface disinfection, may help reduce the long-term dissemination of AMR.

According to the Council of the EU’s recommendation regarding the three AMR targets to be achieved by the EU by 2030, these include reducing the total incidence of bloodstream infections with MRSA, third-generation cephalosporin-resistant *E. coli*, and carbapenem-resistant *K. pneumoniae*, by 15%, 10%, and 5%, respectively, by 2030 against the baseline year 2019 [6]. The results highlighted in our study show that these targets were achieved in the incidence rates registered in our hospital.

Our study has several limitations, related to the small number of strains tested in certain situations and the retrospective nature of the study.

Although the samples collected came from patients hospitalized in one of the largest hospitals in Romania, the fact that the study was conducted in a single hospital may limit the generalizability of the study results. Also, an intensive care unit of a county hospital receives patients with severe pathology, including infectious pathology, which can be caused by resistant pathogens. However, this research represents an important starting point for an extended assessment of the impact of the COVID-19 pandemic on AMR in Romania. Further multicenter studies, including territorial hospitals, could provide more accurate data on the antimicrobial resistance issue in our country.

## 4. Materials and Methods

This research is a retrospective study of all the isolated bacterial pathogens collected from patients admitted to the intensive care unit (ICU) of the Emergency Clinical County Hospital of Craiova, Romania, in pre- and post-COVID-19 pandemic periods. This hospital is a county hospital with 1518 beds (65 beds of ICU) which provides specialized healthcare to patients from Dolj county and the South-West Region of Romania, especially critically ill patients who cannot be treated in lower ranking hospitals. The selected study periods were from 1 April 2019, to 31 March 2020 (pre-COVID-19 period) and 1 July 2021 to 30 June 2022 (post-COVID-19 period). We chose two periods of one year each, with a global decline in cases of SARS-CoV2 infection being registered in the second period. The epidemiological alert period in Romania ended in March 2022.

Data were collected from the clinical pathology databases of the hospital, including samples collected from patients with suggestive signs of infection. Samples collected as part of the hospital’s existing screening protocol were not included. The specimens from all patients were sent to the Hospital’s Laboratory of Microbiology. Samples included blood, urine, sputum/tracheal aspirate (respiratory secretion), pus/wound swabs, exudates, intravascular catheters, cerebrospinal fluid, and sterile fluids. All positive bacterial cultures from patients admitted to the ICU in the studied period were included. Blood samples were collected in specialized bottles provided with the automated system Bact/Alert 70^®^ 3D, harvesting a set of two culture bottles for each patient, including one bottle for aerobic bacteria and one for anaerobic bacteria. Bacterial duplicates, defined as the same pathogen with the same resistance profile isolated from the same patient and the same site of infection, were excluded. Also, samples collected less than 30 days apart, during which the same pathogen was isolated, were excluded. All positive bacterial cultures from patients admitted to the ICU in the studied period were included. The percentage of MDR strains among the clinical isolates from the ICU was analyzed by taking into consideration resistance to at least three different antibiotic groups: aminoglycosides, cephalosporins, carbapenems, tetracyclines, and fluoroquinolones [36].

Multidrug-resistant (MDR) is defined as a pathogen which has acquired non-susceptibility to at least one agent in three or more antimicrobial categories [36].

Pan-drug-resistant (PDR) is defined as a pathogen which has acquired non-susceptibility to all agents tested in the hospital [37].

The identification of the isolated strains and the analysis of the resistance patterns for the action of the appropriate antibiotics were performed using Vitek 2 Compact system [38].

The antimicrobial susceptibility test was carried out according to Clinical Laboratory Standard Institute (CLSI) guidelines [39].

Information about patients’ age, sex, hospital department, sample type, site of infection, and antimicrobial resistance pattern is stored in the Hospital’s Information System. Data were entered and analyzed using Microsoft Excel (2007). Continuous variables like age are expressed as mean ± STDEV (standard deviation). The pattern of micro-organisms was analyzed and expressed as percentages. The percentage of resistant strains was calculated by dividing the resistance strains by the tested ones. The statistical evaluation of possible differences in antibiotic resistance between pre- and post- COVID periods was performed individually for each combination of antibiotics and pathogens, using the chi-square test for independence, or Fisher’s exact test, for small groups. Epi Info software, version 7.2.4.0, was used for all statistical analyses. A two-sided *p*-value ≤ 0.05 was considered to be statistically significant. The authors followed the STROBE Guidelines during the conduct of this research project [40].

## 5. Conclusions

Although for most of the tested antibiotics, no change in the resistance pattern was found, our findings demonstrated, in the post-COVID-19 period, a significantly increasing trend of the antimicrobial resistance rate of Gram-negative pathogens (*Klebsiella* spp. and *Acinetobacter* spp.) against certain antibiotics, including those used only in cases where there are no other therapeutic options. This draws attention to the need to raise awareness among medical staff about the judicious use of antibiotics and to intensify prevention and control measures, leading to a limitation of the circulation and spread of MDR pathogens.

Also, further studies are necessary to provide data on the evolution of AMR, to facilitate the development of appropriate control measures.

## Figures and Tables

**Table 1:** Characteristics of the studied patients hospitalized in the ICU, County Emergency Clinical Hospital Craiova, Romania, pre- and post-COVID-19 era.

Patients		Pre-COVID-19 (No = 1267)	Post-COVID-19 (No = 1354)
Gender	Male	797 (62.90%)	841 (62.11%)
	Female	470 (37.10%)	513 (37.89%)
Age (Mean ± SD)		64 ± 18.60	64 ± 17.51

**Table 2:** Distribution of pathogens isolated from different specimen types in the ICU, County Emergency Clinical Hospital Craiova, Romania, pre- and post-COVID-19 periods (%).

Sample		Species					
		Total	*Acinetobacter* spp.	*E. coli*	*Klebsiella* spp.	*Proteus* spp.	*Pseudomonas* spp.
Blood	Pre-COVID-19	154	6 (3.90%)	17 (11.04%)	4 (2.59%)	7 (4.54%)	85 (55.2%)
	Post-COVID-19	613	29 (4.73%)	35 (5.71%)	5 (0.81%)	9 (1.47%)	246 (40.13%)
Tracheal aspirate/Sputum	Pre-COVID-19	1355	40 (2.95%)	234 (17.27%)	90 (6.64%)	58 (4.28%)	27 (1.99%)
	Post-COVID-19	1294	72 (5.56%)	226 (17.47%)	90 (6.96%)	94 (7.26%)	2 (0.15%)
Pus/wound swabs	Pre-COVID-19	215	23 (10.70%)	32 (14.88%)	27 (12.56%)	19 (8.84%)	10 (4.65%)
	Post-COVID-19	124	13 (10.48%)	25 (20.16%)	16 (12.91%)	10 (8.06%)	6 (4.84%)
Urine	Pre-COVID-19	108	31 (28.70%)	33 (30.56%)	4 (3.70%)	2 (1.85%)	0 (0%)
	Post-COVID-19	115	38 (33.04%)	16 (13.92%)	10 (8.69%)	5 (4.35%)	0 (0%)
Catheters	Pre-COVID-19	10	0 (0%)	1 (10%)	0 (0%)	1 (10%)	0 (0%)
	Post-COVID-19	29	1 (3.44%)	5 (17.24%)	5 (17.24%)	2 (6.90%)	0 (0%)
Other	Pre-COVID-19	98	9 (9.18%)	26 (26.53%)	8 (8.17%)	6 (6.12%)	6 (6.12%)
	Post-COVID-19	106	15 (14.15%)	19 (17.93%)	12 (11.32%)	13 (12.27%)	2 (1.88%)
Total	Pre-COVID-19	1940	109 (5.62%)	343 (17.68%)	133 (6.86%)	93 (4.80%)	128 (6.59%)
	Post-COVID-19	2281	283 (12.41%)	168 (7.36%)	326 (14.92%)	138 (6.05%)	133 (5.83%)
**Sample**		**Species**					
		**CoNS**	***S. aureus***	***Streptococcus* spp.**	***Enterococcus* spp.**	**Other Species**	
Blood	Pre-COVID-19	85 (55.2%)	9 (5.85%)	10 (6.5%)	5 (3.25%)	4 (2.59%)	
	Post-COVID-19	246 (40.13%)	31 (5.05%)	12 (1.96%)	46 (7.5%)	150 (24.47%)	
Tracheal aspirate/Sputum	Pre-COVID-19	27 (1.99%)	407 (30.04%)	263 (19.41%)	0 (0%)	62 (4.58%)	
	Post-COVID-19	2 (0.15%)	364 (28.13%)	173 (13.3%)	38 (2.94%)	34 (2.63%)	
Pus/wound swabs	Pre-COVID-19	10 (4.65%)	49 (22.79%)	4 (1.86%)	8 (3.72%)	21 (9.77%)	
	Post-COVID-19	6 (4.84%)	16 (12.91%)	5 (4.03%)	5 (4.03%)	12 (9.67%)	
Urine	Pre-COVID-19	0 (0%)	1 (0.93%)	1 (0.93%)	26 (24.07%)	9 (8.33%)	
	Post-COVID-19	0 (0%)	0 (0%)	0 (0%)	34 (29.56%)	12 (10.44%)	
Catheters	Pre-COVID-19	0 (0%)	5 (50%)	0 (0%)	0 (0%)	2 (20%)	
	Post-COVID-19	0 (0%)	8 (27.59%)	0 (0%)	2 (6.90%)	2 (6.90%)	
Other	Pre-COVID-19	6 (6.12%)	22 (22.54%)	3 (3.06%)	6 (6.12%)	4 (4.08%)	
	Post-COVID-19	2 (1.88%)	21 (19.81%)	2 (1.88%)	3 (2.83%)	7 (6.61%)	
Total	Pre-COVID-19	128 (6.59%)	493 (25.41%)	281 (14.48%)	45 (2.32%)	102 (5.26%)	
	Post-COVID-19	256 (11.22%)	440 (19.29%)	192 (8.42%)	128 (5.61%)	217 (9.52%)	

**Table 3:** Distribution of pathogens isolated from different specimen types in the ICU, County Emergency Clinical Hospital Craiova, Romania, pre- and post-COVID-19 periods.

	*Klebsiella* spp.			*Escherichia coli*			*Pseudomonas* spp.		
Antimicrobial Agent	Pre-COVID-19 (*n* = 343)	Post-COVID-19 (*n* = 326)	*p* Value	Pre-COVID-19 (*n* = 109)	Post-COVID-19 (*n* = 168)	*p* Value	Pre-COVID-19 (*n* = 93)	Post-COVID-19 (*n* = 133)	*p* Value
Amoxicillin/ clavulanic acid	217 (66.36%)	194 (62.98%)	0.15	31 (29.52%)	60 (36.58%)	0.23	18 (100%)	7 (87.5%)	0.12
Ceftazidime	209 (65.31%)	223 (69.68%)	0.23	32 (31.68%)	64 (38.55%)	0.25	53 (60.92%)	86 (67.72%)	0.30
Ceftriaxone	213 (65.34%)	212 (67.30%)	0.59	37 (35.58%)	55 (33.95%)	0.78	14 (82.35%)	7 (70%)	0.45
Cefotaxime	99 (65.56%)	137 (65.55%)	<0.001 *	7 (26.92%)	40 33.78%	0.31	9 (75%)	9 (81.82%)	0.69
Cefazolin	152 (77.16%)	211 (79.32%)	0.57	32 (57.14%)	62 (51.24%)	0.46	5 (100%)	6 (100%)	-
Cefepime	132 (61.68%)	172 (55.30%)	0.14	21 (33.33%)	40 (25.97%)	0.27	52 (76.47%)	55 (58.51%)	0.01 *
Imipenem	108 (40.60%)	113 (45.56%)	0.25	16 (16.49%)	7 (6.14%)	0.01 *	45 (58.44%)	35 (47.30%)	0.17
Meropenem	111 (45.68%)	106 (44.35%)	0.76	2 (2.60%)	9 (9.57%)	0.06	52 (65%)	47 (57.32%)	0.31
Ciprofloxacin	199 (59.76%)	179 (61.30%)	0.69	43 (39.82%)	64 (43.54%)	0.74	49 (55.68%)	76 (62.29%)	0.33
Levofloxacin	40 (55.56%)	82 (59%)	0.63	13 (52%)	21 (28.76%)	0.03	31 (60.78%)	49 (56.98%)	0.21
Piperacillin/tazobactam	35 (71.43%)	119 (61.34%)	0.19	0 (0%)	24 (24%)	0.14	28 (36.84%)	39 (47.56%)	0.36
Colistin	1 (0.38%)	40 (20.51%)	<0.001 *	3 (4.69%)	25 (32.46%)	<0.001 *	0 (0%)	4 (4.88%)	0.03
Gentamicin	112 (44.62%)	203 (64.85%)	<0.001 *	21 (36.94%)	60 (37.73%)	0.90	38 (61.29%)	77 (60.63%)	0.93
Aztreonam	182 (56.35%)	180 (73.60%)	<0.001 *	27 (27%)	31 (27.43%)	0.94	39 (50%)	34 (39.08%)	0.15
	***Acinetobacter* spp.**				***Proteus* spp.**				
**Antimicrobial** **Agent**	**Pre-COVID-19** **(*n* = 213)**		**Post-COVID-19** **(*n* = 283)**	***p* Value**	**Pre-COVID-19** **(*n* = 133)**		**Post-COVID-19** **(*n* = 138)**	***p* Value**	
Amoxicillin/ clavulanic acid	87 (94.57%)		47 (100%)	0.10	100 (78.13%)		96 (73.85%)	0.42	
Ceftazidime	165 (93.75%)		265 (96.01%)	0.27	89 (72.95%)		100 (73.53%)	0.91	
Ceftriaxone	189 (97.73%)		270 (97.12%)	0.84	99 (76.15%)		91 (67.41%)	0.11	
Cefotaxime	159 (95.78%)		230 (95.43%)	0.86	45 (80.36%)		71 (72.45%)	0.27	
Cefazolin	52 (100%)		49 (100%)	-	80 (94.12%)		103 (84.43%)	0.03 *	
Cefepime	132 (95.65%)		141 (84.43%)	<0.001 *	40 (54.80%)		28 (21.37%)	<0.001 *	
Imipenem	77 (90.58%)		156 (90.17%)	0.91	49 (46.67%)		33 (36.67%)	0.15	
Meropenem	150 (88.76%)		192 (88.48%)	0.93	19 (19.79%)		10 (22.73%)	0.69	
Ciprofloxacin	190 (92.23%)		165 (94.83%)	0.30	82 (68.33%)		93 (75%)	0.24	
Levofloxacin	47 (88.68%)		104 (88.88%)	0.96	20 (86.96%)		41 (75.93%)	0.27	
Piperacillin/ tazobactam	118 (86.76%)		186 (91.62%)	0.14	0 (0%)		20 (26.32%)	0.06	
Colistin	7 (3.37%)		36 (18.09%)	<0.001 *	95 (100%)		56 (100%)	-	
Gentamicin	70 (86.42%)		236 (85.19%)	0.78	72 (76.59%)		75 (55.97%)	0.001 *	
Aztreonam	77 (96.25%)		47 (81.03%)	0.003 *	34 (27.2%)		24 (26.97%)	0.96	

The percentage of each column is calculated by dividing the resistance strains by the tested ones. Samples for which antibiotic resistance testing has not been performed are marked with ‘-’. *p*: *p* value, for comparing between pre- and post-COVID-19 periods; * statistically significant at *p* ≤ 0.05.

**Table 4:** Antimicrobial resistance pattern of Gram-positive bacteria isolated from patients hospitalized in the ICU, County Emergency Clinical Hospital Craiova, Romania, pre- and post-COVID-19 periods.

	*Staphylococcus aureus*			*CoNS*		
Antimicrobial Agent	Pre- COVID-19 (*n* = 493)	Post-COVID-19 (*n* = 440)	*p* Value	Pre-COVID-19 (*n* = 128)	Post-COVID-19 (*n* = 256)	*p* Value
Ciprofloxacin	296 (61.67%)	259 (59.27%)	0.45	90 (70.87%)	149 (59.36%)	0.18
Clindamycin	366 (76.25%)	259 (77.72%)	0.59	85 (66.93%)	163 (68.20%)	0.80
Clarithromycin	219 (56.74%)	243 (60.90%)	0.23	73 (67.60%)	54 (73.97%)	0.35
Doxycycline	190 (40.08%)	135 (51.72%)	0.002 *	52 (54.74%)	7 (43.75%)	0.41
Erythromycin	359 (74.17%)	239 (55.58%)	<0.001 *	100 (80.65%)	99 (68.27%)	0.02 *
Linezolid	1 (0.22%)	8 (3.13%)	<0.001 *	16 (23.88%)	11 (9.65%)	0.009
Penicillin	474 (98.34%)	382 (87.01%)	<0.001 *	112 (93.33%)	231 (93.52%)	0.94
Rifampicin	252 (53.16%)	263 (64.93%)	<0.001 *	57 (45.24%)	157 (62.55%)	0.001 *
Tetracycline	224 (62.57%)	25 (78.12%)	0.07	85 (71.43%)	81 (72.32%)	0.88
Oxacillin	354 (72.99%)	229 (73.40%)	0.89	104 (81.89%)	119 (76.28%)	0.25
Vancomycin	2 (16.67%)	1 (3.58%)	0.14	12 (15.79%)	1 (0.91%)	<0.001 *
Teicoplanin	5 (26.31%)	55 (53.40%)	0.03 *	16 (26.67%)	96 (63.57%)	<0.001 *
	***Streptococcus* spp.**			***Enterococcus* spp.**		
**Antimicrobial** **Agent**	**Pre-COVID-19** **(*n* = 281)**	**Post-COVID-19** **(*n* = 192)**	***p* Value**	**Pre-COVID-19** **(*n* = 45)**	**Post-COVID-19** **(*n* = 128)**	***p* Value**
Ciprofloxacin	5 (100%)	3 (100%)	-	37 (86.05%)	91 (72.8%)	0.07
Clindamycin	80 (29.31%)	50 (27.03%)	0.59	1 (100%)	1 (50%)	-
Clarithromycin	84 (49.12%)	53 (27.03%)	<0.001 *	4 (100%)	5 (71.43%)	0.23
Doxycycline	24 (10.57%)	16 (19.75%)	0.03 *	25 (80.65%)	19 (33.33%)	<0.001 *
Erythromycin	144 (52.55%)	46 (29.11%)	<0.001 *	6 (100%)	42 (75%)	0.16
Linezolid	0 (0%)	0 (0%)	-	2 (4.54%)	1 (1.20%)	0.23
Penicillin	200 (85.11%)	74 (62.18%)	<0.001 *	16 (38.09%)	60 (61.22%)	0.01 *
Rifampicin	4 (20%)	3 (3.61%)	0.008	-	19 (51.35%)	-
Tetracycline	41 (35.04%)	34 (32.69%)	0.71	6 (75%)	13 (41.93%)	0.09
Oxacillin	170 (91.89%)	37 (87.70%)	0.73	-	24 (96%)	-
Vancomycin	3 (1.11%)	0 (0%)	0.26	7 (29.17%)	4 (5.33%)	0.001 *
Teicoplanin	-	-	-	12 (29.27%)	27 (36.98%)	0.40

The percentage of each column is calculated by dividing the resistance strains by the tested ones. Samples for which antibiotic resistance testing has not been performed are marked with ‘-’. *p*: *p* value, for comparing between pre- and post-COVID-19 period; * statistically significant at *p* ≤ 0.05.

## Data Availability

Data is contained within the article.
